# The *SlHsfC1–SlGAI3* Module Controls Tomato Growth and Development via the Gibberellin Signaling Pathway

**DOI:** 10.3390/plants14233617

**Published:** 2025-11-27

**Authors:** Yafei Qin, Mei Wang, Daodao Tang, Lei Ni, Chunyu Shang, Lang Wu, Yu Pan, Jinhua Li, Xingguo Zhang

**Affiliations:** 1 College of Horticulture and Landscape Architecture, Southwest University, Chongqing 400715, China; 2 Key Laboratory of Agricultural Biosafety and Green Production of Upper Yangtze River (Ministry of Education), Southwest University, Chongqing 400715, China; 3 Academy of Agricultural Sciences, Southwest University, Beibei, Chongqing 400715, China

**Keywords:** *SlHsfC1*, dwarf phenotype, gibberellins, exogenous GA_3_, GA signaling pathway, *SlGAI3*

## Abstract

In agricultural production, plants commonly suppress their growth and development under abiotic stresses. We identified the heat shock transcription factor *SlHsfC1*, and overexpression (OE) lines resulted in a dwarf phenotype. Overexpression lines exhibited reduced cell size and elevated levels of bioactive gibberellins (GAs). However, applying external GA_3_ did not restore the dwarf phenotype. Gene expression analysis showed that GA biosynthesis pathway genes (*SlKO*, *SlKAO*, *SlGA20ox3*, and *SlGA20ox4*) were upregulated, whereas GA metabolic pathway genes (*SlGA2ox1* and *SlGA2ox2*) were downregulated, leading to the accumulation of bioactive gibberellins. However, GA signal transduction pathway genes (*SlGAI2* and *SlGAI3*) were also upregulated, thereby impairing gibberellin signaling in these lines. Protein–DNA interaction assays confirmed that *SlHsfC1* directly binds the *SlGAI3* promoter and activates its expression. Thus, *SlHsfC1* regulates plant height by modulating key genes in the gibberellin signaling pathway.

## 1. Introduction

Under abiotic stress conditions, plants often limit their growth and development as a survival mechanism. Earlier research has demonstrated that plant dwarfing can enhance resistance to various stresses [1,2]. Plant dwarfing can result from various factors, among which hormonal regulation plays a predominant role.

Certain gibberellins (GAs), such as GA_1_, GA_3_, GA_4_, and GA_7_, are essential for promoting plant development by modulating diverse physiological processes [3,4,5]. These active GA compounds are synthesized via a multistep biosynthetic pathway that begins with the precursor geranylgeranyl diphosphate (*GGPP*). The initial steps involve its transformation into ent-kaurene, a reaction catalyzed by ent-copalyl diphosphate synthase (*CPS*) and ent-kaurene synthase (*KS*) [4]. Subsequently, ent-kaurene is oxidized to form GA12, an intermediate, through the enzymatic actions of ent-kaurene oxidase (*KO*) and ent-kaurenoic acid oxidase (*KAO*). The progression from GA12 to the bioactive forms of gibberellins is mediated by GA20-oxidase (*GA20ox*) and GA3-oxidase (*GA3ox*). Mutations or functional disruptions in genes encoding these enzymes—such as *GA20ox1* in Arabidopsis, *OsGA20ox2* and *OsGA20ox3* in rice, or *GA3ox1* in Arabidopsis, *OsGA3ox2* in rice, and *ZmGA3ox2* in maize—commonly lead to dwarfism due to impaired GA signaling [6,7,8,9,10,11,12,13]. GA2ox enzymes, which deactivate bioactive gibberellins, have been well characterized in *Arabidopsis* and rice, clarifying their role in GA catabolism and homeostasis [14,15,16,17,18,19,20].

GA perception and signaling are mediated by the GA–GID1–DELLA module, the central regulator of the GA pathway [21]. At low GA levels, GID1 remains inactive and does not bind downstream effectors. When GA levels rise, the hormone binds to GID1, inducing a conformational rearrangement that facilitates its association with DELLA proteins. This binding leads to the assembly of the GA–GID1–DELLA complex. The DELLA repressors are then targeted for degradation by the Skp1–Cullin–F-box (SCF) E3 ubiquitin ligase, which tags them with polyubiquitin chains [22]. Ubiquitinated DELLA proteins are degraded by the 26S proteasome, releasing GA-responsive gene expression. As negative regulators of GA signaling, DELLA proteins inhibit growth related processes. Their proteasomal degradation is necessary for activating GA-induced developmental responses. Alterations or mutations that abolish or reduce the function of *DELLA* genes frequently result in dwarfism, underscoring their key role in restricting growth [23,24].

Heat shock transcription factors (Hsfs) are broadly distributed among eukaryotic organisms, with plants exhibiting a notably greater number of Hsf genes compared to animals. For instance, only six Hsf isoforms have been identified in the human genome [25]. *Arabidopsis thaliana* contains 21 Hsf genes [26], *Oryza sativa* has 25 [26], *Solanum lycopersicum* encodes 24 [27], *Triticum aestivum* possesses 56 [28], and *Zea mays* contains 30 Hsf members [29]. The Hsf protein family is defined by conserved structural motifs. They possess a DNA-binding domain, an HR-A/B oligomerization domain, and nuclear localization signals. The DBD recognizes specific cis-elements such as the heat shock element (HSE, -GAAnnTTC-) and the stress response element (STRE, -AGGGG-), with HSE binding being especially prominent in HSFA1 proteins. Hsfs are grouped into classes A, B, and C by OD structure; class A and C differ by HR-A/B linker lengths of 21 and 7 amino acids, respectively [30]. Additionally, class A Hsfs harbor an AHA activation domain that stimulates transcription [28], whereas class B Hsfs are marked by a conserved -LFGV- motif proximal to their NLS domain [29].

Although class C Hsfs are not well characterized, they likely enhance heat tolerance by activating heat-responsive genes [31]. In this study, to investigate the function of *SlHsfC1*, we generated overexpression lines and CRISPR-induced (CR) knockout lines and observed a dwarf phenotype in the overexpression lines. Exogenous GA_3_ treatment failed to alleviate these abnormalities or restore normal plant development. Measurement of gibberellin content suggested that the overexpression lines no longer exhibited GA deficiency. Our findings suggest that *SlHsfC1* can regulate *SlGAI3*, a member of the DELLA protein family involved in gibberellin signaling. This regulation may underlie the observed dwarfism and developmental abnormalities, since DELLA proteins repress GA responses and thereby contribute to gibberellin insensitivity in affected tomato plants. This study reveals that *SlHsfC1* likely participates in regulating plant height by modulating GA signaling.

## 2. Materials and Methods

### 2.1. Plant Materials, Growth Conditions, and Treatments

In this study, overexpression (OE6 and OE12) and CRISPR lines (CR16 and CR41) with the AC cultivar as the genetic background were used. Tomato plants of the AC cultivar were genetically engineered to either overexpress *SlHsfC1* (OE6, OE12) or carry CRISPR/Cas9-induced knockouts (CR16, CR41) for functional analyses. The plants were cultivated in a controlled growth chamber at 25 ± 2 °C under a photoperiod of 16 h light and 8 h dark.

To analyze the hormone-responsive expression pattern of *SlHsfC1*, tomato plants at the five-true-leaf stage were treated with various phytohormones by foliar spraying, including 1% (*v*/*v*) ethylene (ETH), 100 μM gibberellic acid (GA_3_), and 100 μM jasmonic acid (JA), 100 μM Salicylic acid (SA), with water as control [32].

To determine whether the dwarf phenotype is GA-dependent, overexpression plants (OE6, OE12), CRISPR lines (CR16 and CR41) and wild-type plants were treated with 100 μM GA_3_ (in 0.02% Tween-20) every three days, starting from germination and one-month-old seedings. The control group consisted of plants sprayed with an identical volume of solvent, which was a solution containing 0.02% Tween-20. Plant height was recorded following each spraying treatment.

### 2.2. Generation of Transgenic Lines

To generate *SlHsfC1* overexpression lines, *SlHsfC1* (Solyc12g007070) CDS was amplified using PrimeSTAR^®^ MAX (Takara, Shiga, Japan), with primers in Supplementary Table S1. The amplified fragment was cloned into pBinary by homologous recombination. The *SlHsfC1*-targeting gRNA was amplified from p043 and cloned into pKSE401 for CRISPR editing [33]. Using recombination cloning per manufacturer’s protocol. Constructs were transformed into *A. tumefaciens* LBA4404 by freeze–thaw and used for AC tomato leaf disc transformation [34].

### 2.3. Subcellular Localization Analysis

*SlHsfC1* CDS (without stop codon) was cloned into pBinary 35S-Flag-GFP. These constructs were introduced into *Nicotiana benthamiana* leaves via transient transformation using *Agrobacterium tumefaciens* GV3101. After 48 h of incubation in darkness, GFP signals were observed using a Zeiss LSM 780 confocal microscope.

### 2.4. Tissue Sectioning and Cell Morphology Quantification

Wild-type and overexpression lines were grown until the development of five true leaves, and then entire seedlings were sent to Servicebio for tissue sectioning. Cellular morphology and size were subsequently examined using light microscopy. The number of cells and the average cell size were quantified using ImageJ software 1.54g based on microscopic images of tissue sections.

### 2.5. Measurement of Gibberellin Content

When wild-type, overexpression plants (OE6, OE12), and knockout plant CR16, CR41 reached the stage of five true leaves, the top three true leaves (from top to bottom) were collected and sent to Covinced-Test (https://www.woyaoce.cn/) for gibberellin measurement.

### 2.6. RNA-Seq and RT-PCR

Total RNA was extracted from leaves of wild-type AC, overexpression (OE6), and CRISPR lines (CR16) grown at 25 °C. For RNA-Seq, three biological replicates were prepared, and library construction and sequencing were performed by Gene Denovo (Guangzhou, China) on the Illumina (San Diego, CA, USA) high-throughput sequencing platform with a sequencing depth of 10×. Differentially expressed genes were identified using a threshold of |log_2_FC| > 1 and FDR < 0.05. For quantitative real-time PCR (RT–qPCR), cDNA was synthesized from total RNA using ExonScript RT Mix (Bioground, Chongqing, China). qPCR primers were designed with QuantPrime (https://quantprime.mpimp-golm.mpg.de (accessed on 15 November 2025)). SYBR Green RT–qPCR was performed on qTOWER 2.0 (Analytik Jena, Jena, Germany) following an established protocol [35]. *SlElfα* (Solyc11g005330) was used as reference; relative expression was calculated by 2^^−ΔΔCt^ [36].

### 2.7. Yeast One-Hybrid Assay

*SlHsfC1* CDS was cloned into pGADT7 to create the AD-*SlHsfC1* prey vector. A 600 bp *SlGAI3* promoter fragment was amplified with specific primers. The promoter was cloned into pAbAi by homologous recombination to create pAbAi-*SlGAI3*pro. The pAbAi-*SlGAI3*pro vector was linearized using *Bbs*I and transformed into Y1HGold cells via the LiAC-PEG method, followed by selection on SD/-Ura medium. After establishing AbA screening conditions, pGADT7-*SlHsfC1* was transformed into Y1HGold (pAbAi-*SlGAI3*pro) using the same method. Transformants were selected on SD/-Ura/-Leu medium with optimal AbA concentration. Positive clones were cultured until the OD_600_ reached 0.6, then diluted in 0.9% NaCl to 10-fold, 100-fold, and 1000-fold concentrations. The dilutions were subsequently spotted onto SD/-Ura/-Leu selective medium supplemented with the suitable AbA.

### 2.8. Transactivation Assay

To determine whether *SlHsfC1* regulates the expression of *SlGAI3*, a transient dual-luciferase assay was performed in *Nicotiana benthamiana*. The *SlHsfC1* CDS was cloned into pGreenII 62-SK, and a 600 bp *SlGAI3* promoter fragment into pGreenII 0800-LUC, generating the effector and reporter constructs, respectively. Both constructs were transformed into *A. tumefaciens* GV3101 (pSoup). The reporter was co-infiltrated with either the effector or empty pGreenII 62-SK control into *N. benthamiana* leaves. Infiltrated plants were incubated in dark at 25 °C for 48 h. Fluorescein potassium salt was subsequently applied to the leaf surface, and firefly luciferase signals were visualized using an in vivo imaging system. Dual-luciferase activity was quantified with the Cytation 5 system (BioTek, Shoreline, WA, USA) to evaluate the regulatory impact of *SlHsfC1* on *SlGAI3* promoter activity.

### 2.9. Electrophoretic Mobility Shift Assay

EMSA was used to test SlHsfC1 protein binding to the HSE motif in the *SlGAI3* promoter. The coding region of *SlHsfC1*, excluding the stop codon, was inserted into the pET-32a vector to create the pET-32a-*SlHsfC1* expression construct. The method for prokaryotic expression of the protein was examined by [37]. The His-tagged SlHsfC1 protein was then purified for use in EMSA binding assays. A 21 bp biotin-labeled DNA probe containing the HSE element (GAAnnTTC) from the *SlGAI3* promoter was synthesized by Tsingke Biotechnology (Tsingke Biotechnology Co., Ltd., Beijing, China). In parallel, an unlabeled (cold) probe and a mutant probe in which the HSE element was replaced with a non-specific sequence (AAAAAAAAA) were also synthesized to serve as competitors and specificity controls in EMSA. The EMSA system was configured according to the instructions provided with the EMSA kit (Beyotime Biotechnology, Shanghai, China), The nylon membrane was exposed using FluorChem M system (ProteinSimple, San Jose, CA, USA) to visualize the shifted DNA-protein complexes.

### 2.10. Chromatin Immunoprecipitation (ChIP) Qpcr Assay

Transgenic tomato plants expressing Flag-*SlHsfC1*-eGFP and Flag-eGFP were cultivated for 30 days. Leaves were collected and subjected to ChIP following the method described in [38]. After enrichment of DNA, qPCR was performed using primers specific to the *SlGAI3* promoter region.

### 2.11. Data Statistics and Analysis

Experiments were conducted with ≥3 biological replicates; significance was evaluated using Student’s *t*-test, two-way ANOVA as appropriate. Data were analyzed and plotted in GraphPad Prism 9.0; error bars denote mean ± SD.

## 3. Results

### 3.1. Origin and Protein Structure Analysis of HsfC Gene

**Figure 1:**
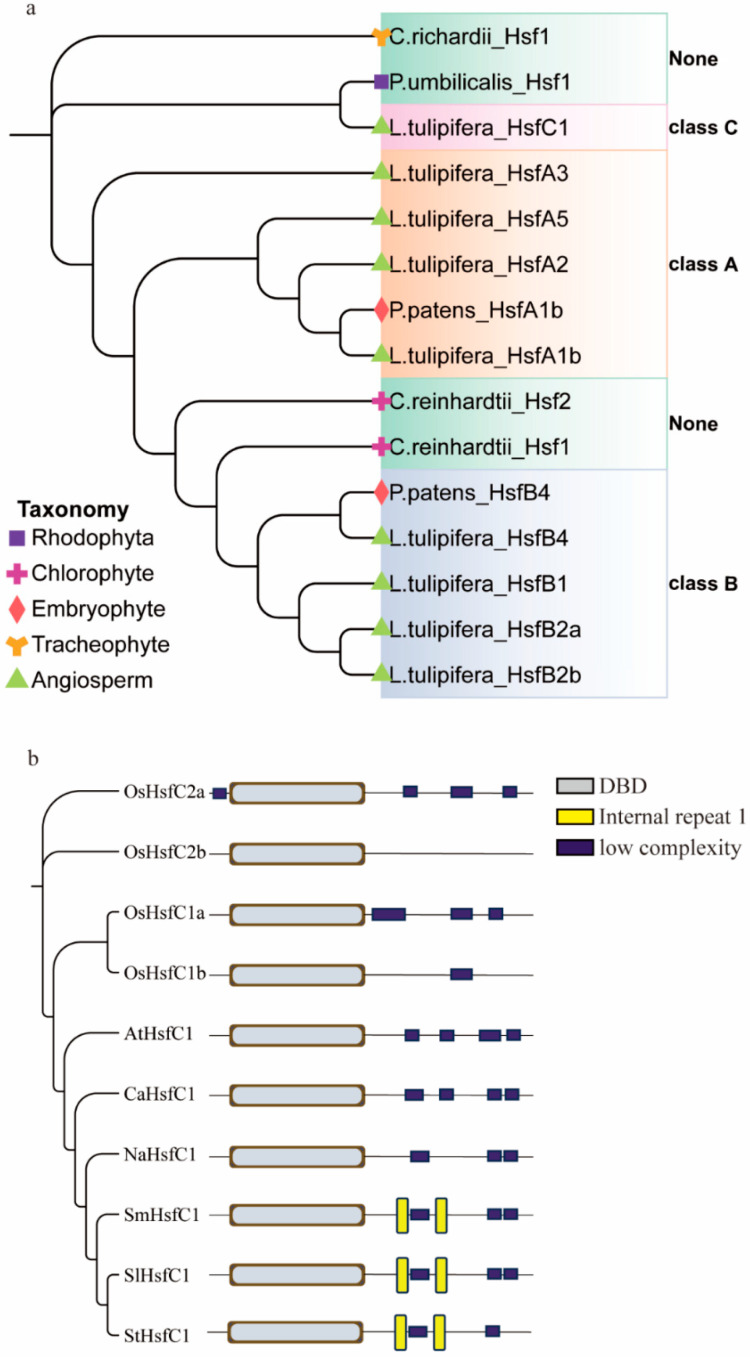
Evolutionary analysis of tomato heat stress factor *SlHsfC1*. (**a**) Phylogenetic relationships between Hsf family from *Porphyra umbilicalis*, *Chlamydomonas reinhardtii*, *Physcomitrium patens*, *Liriodendron tulipifera*. (**b**) Phylogenetic relationships between *SlHsfC1* (Solyc12g007070) and homologous HsfC from *Arabidopsis*, tomato, *Oryza sativa*, *Nicotiana benthamiana*, pepper, potato, eggplant.

The Hsf family genes are present in primitive eukaryotes of the Rhodophyta, with only one Hsf gene identified in *Porphyra umbilicalis* (*PumHsf1*). In the algae *Chlamydomonas reinhardtii*, two Hsf genes, *CreHsf1* and *CreHsf2*, are present. These genes later diverged into the Hsf A and Hsf B subfamilies. Similarly, in the embryophyte *Physcomitrium patens*, the genes *PpaHsfA1b* and *PpaHsfB4* represent these subfamilies. Angiosperms (*Liriodendron tulipifera*) are notable for being the first to develop Hsf C subfamily genes, along with more extensive Hsf A and Hsf B gene repertoires. Evolutionarily, it appears that C-class Hsf subfamily genes evolved from Hsf A subfamily genes. (Figure 1a).

Both monocot and dicot plants harbor Hsf C subfamily genes. Specifically, *Oryza sativa* L, *Arabidopsis thaliana* L, *Solanum melongena* L, *Capsicum annuum* L, *Nicotiana benthamiana*, *Solanum tuberosum* L, and *Solanum lycopersicum* L. each possess one Hsf C gene, whereas rice harbors four. The secondary structures of the proteins encoded by these Hsf C genes are relatively simple, comprising the DBD, OD, and NLS, which are characteristic of all Hsf family genes. In addition to these conserved domains, Hsf C genes further exhibit low-complexity protein sequences and repetitive sequences (Figure 1b). HsfC originates from angiosperms, and there are double-ended repeats in the HsfC gene in the Solanaceae family.

### 3.2. Subcellular Localization and Hormone Expression Patterns of SlHsfC1

**Figure 2:**
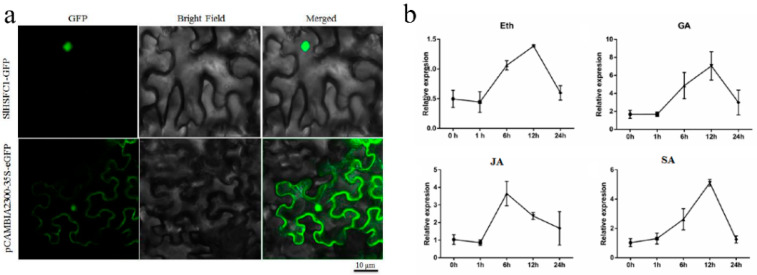
Subcellular localization and hormone expression patterns of *SlHsfC1*. (**a**) Nuclear localization of *SlHsfC1* in *Nicotiana benthamiana* leaves. Bars = 10 μm. (**b**) *SlHsfC1* responds to gibberellin, salicylic acid, abscisic acid, and ethylene hormonal signals.

*SlHsfC1* was transiently expressed in tobacco leaves using the pBinary 35S-*SlHsfC1*-eGFP construct and was localized predominantly in the nucleus, whereas the control vector pbinary 35S-eGFP was localized in the cytoplasmic nucleus (Figure 2a). Furthermore, *SlHsfC1* was found to respond to treatments with gibberellin, salicylic acid, jasmonic acid, and ethylene (Figure 2b).

### 3.3. SlHsfC1 Influences Tomato Growth and Developmental Processes

**Figure 3:**
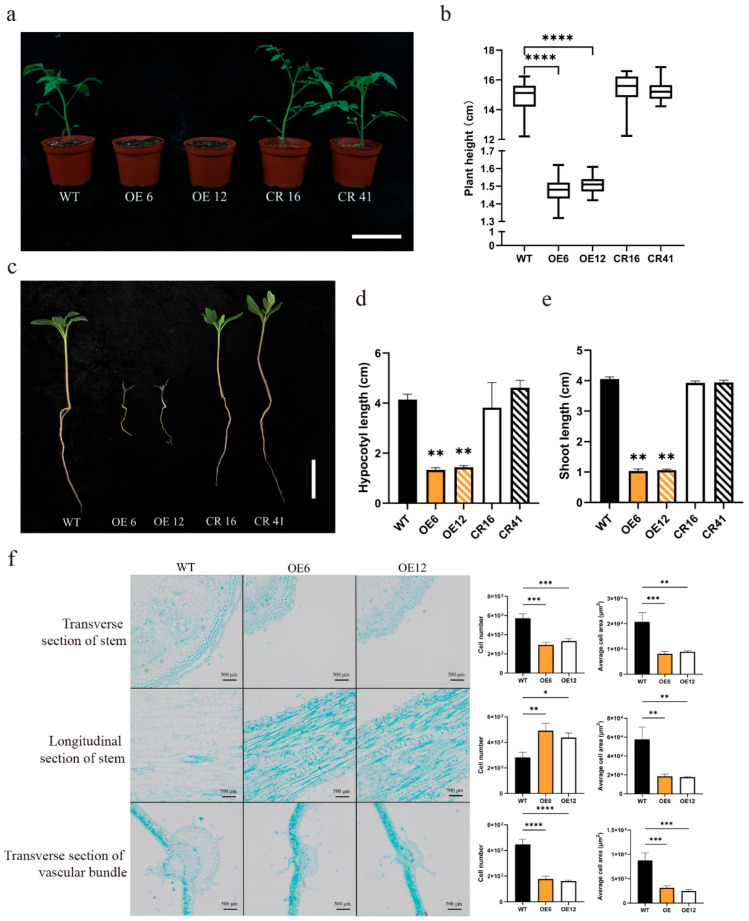
Overexpression of *SlHsfC1* inhibits tomato plant growth. (**a**) Phenotypes of one-month-old seedlings of wild type (AC), overexpression lines (OE6, OE12), and CRISPR knockout lines (CR16, CR41). Scale bar = 10 cm. (**b**) Plant height measurements at one month of age. Statistical analysis by Student’s *t*-test (n = 15). (**c**) Hypocotyl morphology of two-week-old seedlings from overexpression lines (OE6, OE12), CRISPR lines (CR16, CR41), and wild-type (AC). Scale bar = 1 cm. Data analyzed using student *t*-test (n = 3). (**d**) Quantification of plant height corresponding to panel (**c**). (**e**) Measurement of hypocotyl length in seedlings shown in (**c**). (**f**) Microscopic analysis of stem tissue sections from WT, OE6, and OE12 plants, including quantification of cell number and average cell size. Data are expressed as mean ± standard deviation. Statistical significance is indicated as follows: * *p* < 0.05; ** *p* < 0.01; *** *p* < 0.001; and **** *p* < 0.0001.

Overexpression of the *SlHsfC1* gene severely impairs plant development at the seedling stage, leading to significant morphological abnormalities. In contrast, no significant morphological differences were observed between the *SlHsfC1* knockout strain and the wild-type AC. At 30 days post-germination, the *SlHsfC1* overexpression plants showed a plant height of 1.5 to 2.0 cm compared to the wild-type AC and *SlHsfC1* knockout strains, which range from 13 to 18 cm (Figure 3a,b). Additionally, the root system of the *SlHsfC1* overexpression plants was poorly developed, with a root length of approximately 1.5 cm, while the wild-type AC and knockout strains had root lengths of 3 to 4 cm. Meanwhile, shoot lengths showed similar results to root lengths (Figure 3c–e). Cross-sections of stems and leaves revealed that, compared to the wild-type AC, overexpression of *SlHsfC1* led to both a reduction in cell number and a decrease in average cell size. However, in longitudinal sections of the stem, the *SlHsfC1* overexpression plants exhibited an increase in cell number, while the average cell size remained reduced (Figure 3f). The growth of overexpressing plants was abnormal at the seedling stage, the growth of both the aboveground and underground parts was hindered, and the average cell size was reduced.

### 3.4. Exogenous GA3 Treatment Did Not Recover Growth, and SlHsfC1 Overexpressors Showed Increased GA Content Relative to Wild Type

**Figure 4:**
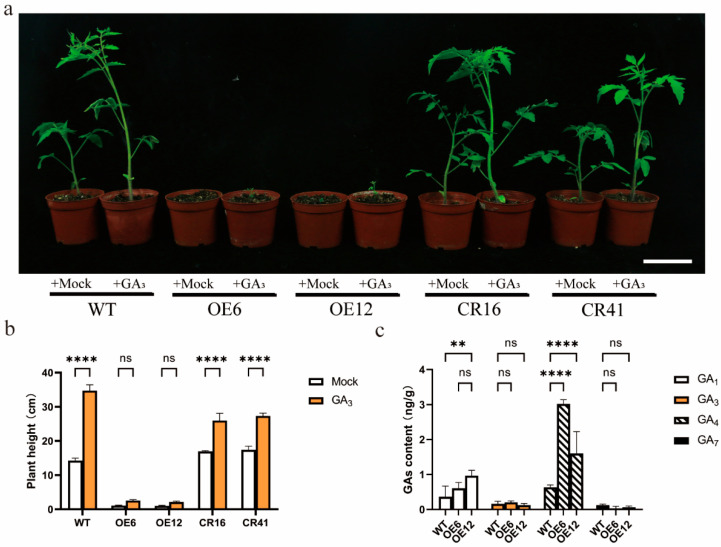
Dwarfing of *SlHsfC1* overexpressing plants was not caused by GA deficiency. (**a**) Phenotypic comparison of *SlHsfC1*-overexpressing lines, CRISPR-Cas9 knockout lines (CR16, CR41), and wild-type (AC) plants under GA_3_ treatment (+GA_3_) or mock treatment (+mock). Spray once every three days for a month. Scale bar  =  10 cm. (**b**) Heights of plants shown in (**a**). Exogenous application of GA_3_ influences various aspects of overexpression lines (OE6, OE12), Crispr lines (CR16, CR41), and wild-type (AC) plant development. Data analyzed using two-way ANOVA (n = 3) (**c**) Quantification of gibberellin levels in *SlHsfC1* overexpression lines. Data analyzed using two-way ANOVA (n = 3). ** *p*  <  0.01, **** *p*  <  0.0001; ns, not significant.

Exogenous application of IAA failed to rescue the dwarf phenotype of *SlHsfC1*-overexpressing plants (Figure S1). After GA_3_ treatment, the overexpressing strain did not recover normal growth, and the increase in plant height was insignificant (Figure 4a,b). In contrast, both the wild-type and knockout strains exhibited a significant increase in height following GA_3_ application (Figure 4a,b). Interestingly, the active gibberellin content in the overexpressing lines remained unchanged. Instead, GA_1_ and GA_4_ levels were higher in the overexpressing lines compared to the wild type (Figure 4c). Additionally, the knockout strain showed a marked increase in GA_1_ and GA_3_ levels relative to the wild type (Figure S3). Spraying GA_3_ could not solve the problem of dwarfing in overexpressed plants, and the active GA in overexpressed plants was not reduced.

### 3.5. Prolonged Application of GA3 Might Not Restore Growth and Development

**Figure 5:**
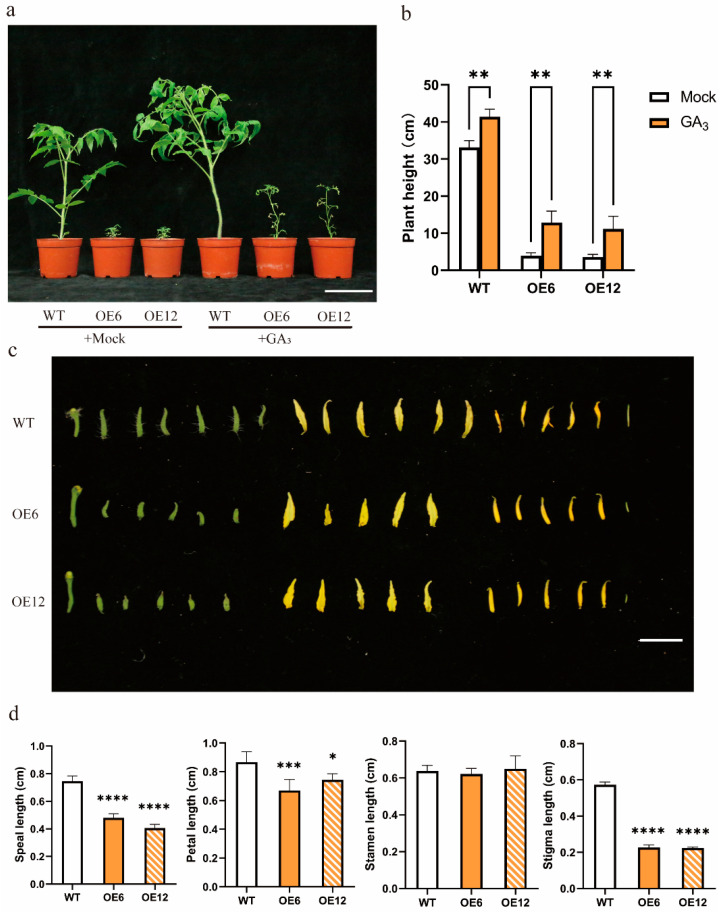
Long-term treatment with GA_3_ in *SlHsfC1* overexpression lines led to altered plant architecture and floral organ abnormalities. (**a**) Morphological comparison of *SlHsfC1* overexpression lines, CRISPR-Cas9 knockout lines (CR16, CR41), and wild-type (AC) plants subjected to either 100 μM exogenous GA_3_ (+GA_3_) or mock treatment (+mock). Spray once every three days for two months. Scale bar  =  10 cm. (**b**) Quantification of plant height corresponding to the plants shown in (**a**). significance by two-way ANOVA (n = 3): ** *p*  <  0.01. (**c**) Floral organ morphology in wild-type and overexpression lines. Scale bar  =  1 cm. (**d**) Measurement of floral organ height corresponding to (**c**). Data are shown as mean  ±  SD (n = 3); significance by Student’s *t*-test: * *p*  <  0.05, *** *p*  <  0.001, **** *p*  <  0.0001.

After 60 days of GA_3_ treatment from seed germination, the overexpressing plants grew taller, but the stems were slender, the leaves were still small, and the growth was thin (Figure 5a). After spraying GA_3_ continuously for 6 months, the plants were able to flower, additionally, the flowers of the overexpression plants are smaller than those of the wild-type AC (Figure 5b). As a result of long-term spraying of GA_3_, parthenogenesis is triggered, and the fruit has no seeds (Figure S3). In field cultivation, the average high temperature from June to August in Chongqing, China, was 38 °C, and it even reached more than 40 °C; the wild-type AC could not grow and develop in early July, and overexpression plants could not flower and set fruit normally in mid-June (the temperature in mid-June was 35–37 °C). Long-term application of GA could not restore the growth of overexpressing plants, and overexpressing plants could not survive the summer.

### 3.6. SlHsfC1 Affects GA Synthesis Pathway and Signal Transduction Pathway

**Figure 6:**
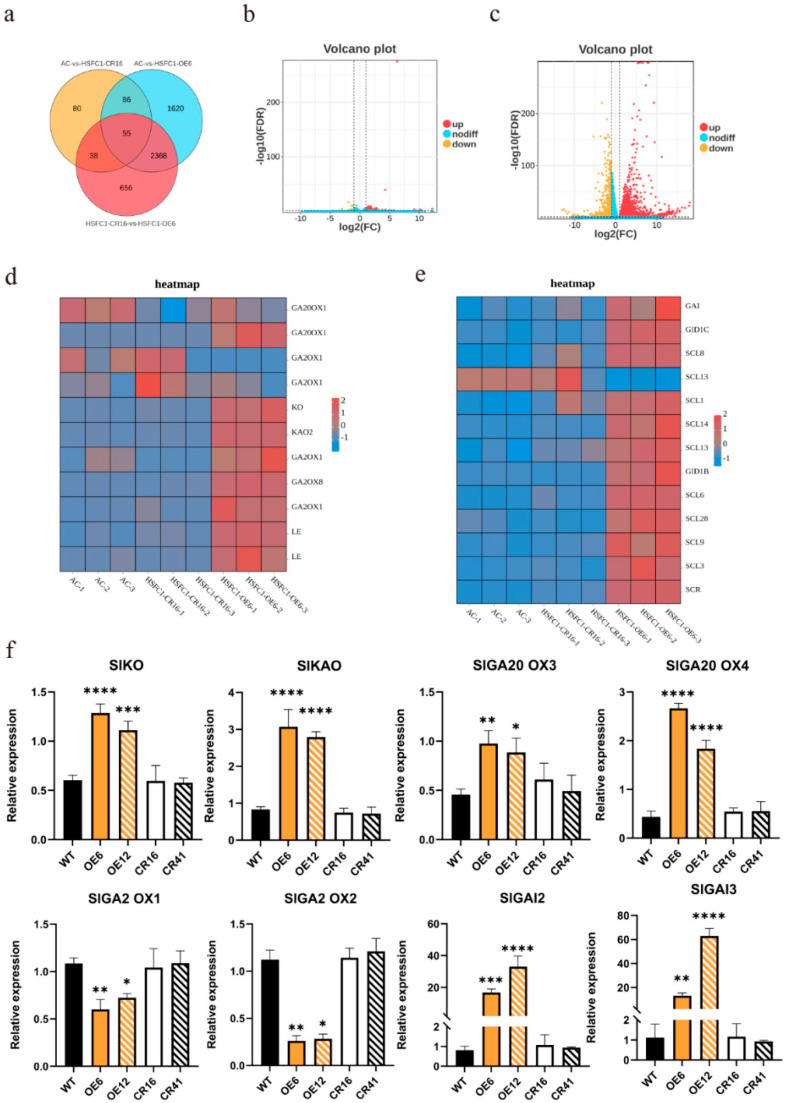
Genes of the GA pathway expression in overexpressing lines, Crispr lines and wild type. (**a**) Venn diagram showing differentially expressed genes (DEGs) among wild-type (AC), *SlHsfC1*-overexpressing lines (OE6, OE12), and CRISPR-Cas9 knockout lines (CR16, CR41) based on RNA-Seq analysis. (**b**,**c**) Volcano plots showing differentially expressed genes (DEGs) in transcriptome comparisons between wild-type (AC) and *SlHsfC1*-overexpressing lines (OE6, OE12), as well as between wild-type and CRISPR-Cas9 knockout lines (CR16, CR41). Significantly upregulated genes (|log_2_FC| > 1, FDR < 0.05) are highlighted in red, downregulated genes in brown, and non-significant genes in blue. (**d**) Expression of gibberellin biosynthesis genes of RNA-Seq suggest that key genes. (**e**) Expression of gibberellin signal genes of RNA-Seq suggest that key genes. (**f**) RT–qPCR analysis validated the RNA-Seq results, confirming the altered expression of key genes involved in GA biosynthesis and GA signal transduction. Statistical significance was determined by Student’s *t*-test (n = 3): * *p*  <  0.05, ** *p*  <  0.01, *** *p*  <  0.001, **** *p*  <  0.0001.

RNA-Seq revealed significant gene expression changes in overexpression and knockout lines relative to wild type. In the overexpression lines, 3586 genes were upregulated, and 543 were downregulated compared to the wild type. The knockout line exhibited 233 upregulated and 26 downregulated genes (Figure 6a–c). Among them, the differentially expressed genes were primarily enriched in pathways related to abiotic stress responses and biological regulation (Figure S4). Notably, 141 differentially expressed genes were common to both overexpression and knockout lines. These differentially expressed genes are primarily enriched in biological processes related to biosynthesis and stress resistance. In the overexpression strain, genes involved in gibberellin biosynthesis, such as *SlKO*, *SlKAO2*, and *SlGA20ox*, were upregulated. Similarly, genes related to the GA signaling response, including *SlGAI3*, *SlGID1C*, and *SlGID1B*, were also upregulated (Figure 6d–f). In overexpression lines, GA synthesis genes were upregulated, GA degradation genes were downregulated, and GA signal transduction genes were upregulated.

### 3.7. SlGAI3 Is a Direct Target Gene of SlHsfC1

**Figure 7:**
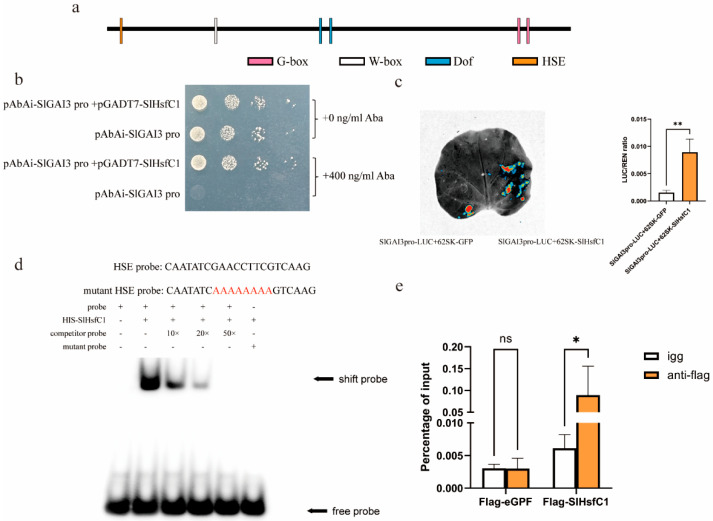
SlHsfC1 protein binds to the HSE element on the *SlGAI3* promoter and activates the transcription of *SlGAI3*. (**a**) Analysis of Cis-Acting Elements on the *SlGAI3* promoter. (**b**) The pAbAi-*SlGAI3*pro and pGADT7-*SlHsfC1* plasmids were co-introduced into Y1HGold yeast cells, which were subsequently grown on SD/-Leu/-Ura agar supplemented with 400 ng/mL aureobasidin A (AbA) for selection. (**c**) The dual-luciferase assay, pGreen0800-*SlGAI3*pro and pGreen62SK-*SlHsfC1* were transiently co-transformed into *Nicotiana benthamiana* leaves. The co-infiltration of pGreen0800-*SlGAI3*pro with empty vector pGreen62SK served as the control. Statistical significance was assessed using Student’s *t*-test (n = 3). ** *p*  <  0.01. (**d**) EMSA assay. The biotin-labeled probe (biotin-GAANNTTC-) served as the experimental probe, while cold competitor probes (-GAANNTTC-) were added at 10×, 20×, and 50× molar excess to assess binding specificity. A mutant probe (biotin-AAAAAAAA-) was included as a negative control. (**e**) ChIP-qPCR of Flag-SlHsfC1 and *SlGAI3* promoter. Statistical significance was assessed using two-way ANOVA (n = 3). * *p*  <  0.05.

The promoter of *SlGAI3* contains several cis-acting elements, including HSE, CGCG box, Dof, W-box, and G-box (Figure 7a). Notably, HSE was located at −550 bp within the promoter. At a concentration of 400 ng/mL of AbA, the pabai-*SlGAI3* pro no longer exhibited autoreactivating activity. At the same concentration, the pGADT7-*SlHsfC1* and pabai-*SlGAI3* promoter bind to each other (Figure 7b). Furthermore, co-transient infiltration of tobacco leaves with pGreen 62SK-*SlHsfC1* and pGreen 0800-*SlGAI* promoter produced a strong signal, whereas no signal was detected in the negative control (Figure 7c). In an EMSA assay, the *SlHsfC1* protein was shown to bind specifically to the “GAAnnTTC” sequence on the *SlGAI3* promoter. However, this binding ability was lost after mutation of the “GAAnnTTC” sequence (Figure 7d). ChIP-qPCR analysis showed that the enrichment of the *SlGAI3* promoter fragment by Flag-*SlHsfC1*-eGFP was significantly higher in the in vivo experimental group compared with the IgG control group. In contrast, no significant difference in enrichment was observed for Flag-eGFP among the negative control plants (Figure 7e). *SlGAI3* is the target gene of *SlHsfC1*, and its up-regulation may be the cause of *SlHsfC1* overexpression leading to dwarfing of transgenic plants.

## 4. Discussion

### 4.1. Overexpression of SlHsfC1 Results Dwarf Phenotype but Not Due to GA Deficiency

Plant height is a key trait influencing crop architecture, land use efficiency, nutrient allocation, and overall management strategies in agricultural systems [39,40]. In this study, we demonstrate that the heat shock transcription factor *SlHsfC1* modulates tomato plant height by influencing the gibberellin signaling pathway.

The *SlHsfC1* overexpression lines displayed a distinct dwarf phenotype (Figure 3a–e). Plant height depends on both internode number and length [41,42]. Further analyses demonstrated that *SlHsfC1* impacts plant height through regulation of cell size and cell numbers (Figure 3f), a common effect linked to gibberellin activity [43]. CRISPR mutants displayed no height differences from wild type, possibly due to *SlHsfC1* overexpression disrupting GA signaling. Protein accumulation within the gibberellin signaling pathway often leads to reduced plant height, and mutations affecting key enzymes can cause pronounced dwarfism and growth retardation [44,45]. Typically, defects in GA biosynthesis can be mitigated by the application of exogenous GA [43,46], with affected plants generally exhibiting lower levels of bioactive GAs such as GA_1_ and GA_4_ compared to wild-type controls [47]. To test whether external GA_3_ could alleviate the dwarf phenotype observed in *SlHsfC1* overexpression lines, we treated plants with GA_3_. However, exogenous GA_3_ failed to restore normal growth in these transgenic lines (Figure 4a,b). This observation contradicts the hypothesis that dwarfism in the overexpression lines is due to GAs deficiency, especially since elevated levels of GA_1_ and GA_3_ were detected in these plants (Figure 4c). Given that GA_3_ supplementation did not rescue growth, it is likely that the dwarf phenotype arises from disrupted GA signaling rather than a shortage of GA biosynthesis [48,49]. Furthermore, excessive GA accumulation might influence plant height through pathways independent of GA signaling mechanisms [50,51].

We examined the expression of components in the gibberellin signaling pathway in transgenic lines using RT–qPCR and RNA-Seq analyses to pinpoint possible target genes of *SlHsfC1*. In the overexpression lines, 3586 genes were found to be significantly upregulated (Figure 6a), including numerous genes associated with GA biosynthesis and signal transduction (Figures S4 and S5). Additionally, several stress-responsive genes exhibited differential expression. As an Hsf family member, *SlHsfC1* aligns with recent findings indicating that HsfC-type proteins contribute to improved heat tolerance [31,52,53]. Our data support emerging evidence that stress-responsive genes may negatively impact plant growth and development [1,54,55]. Notably, transcripts of *SlKO*, *SlKAO*, *SlGA20ox3*, and *SlGA20ox4* were elevated in the overexpressing plants (Figure 6a). KO oxidizes ent-kaurene to ent-kaurenoic acid, which KAO converts to GA_12_, a key gibberellin biosynthesis step [56]. *GA20ox* and *GA3ox* mediate bioactive GA synthesis from GA_12_; *GA20ox* mutations often cause GA-responsive dwarfism [6,57,58]. In contrast, *GA2ox1* and *GA20ox2*, which encode enzymes responsible for inactivating bioactive GAs by converting them or their precursors into inactive forms, were downregulated in the overexpression lines [59]. *SlGAI2* and *SlGAI3*, encoding DELLA repressors of GA signaling, were also upregulated. GA binding triggers GID1 conformational changes, promoting GA–GID1–DELLA complex formation. DELLA proteins are polyubiquitinated by SCF/GID2 and degraded by the 26S proteasome, relieving repression on GA-responsive genes [60,61,62]. Excessive accumulation of DELLA proteins, however, causes severe dwarfism not restored by exogenous GA [63,64], which may explain the persistent dwarf phenotype observed in *SlHsfC1* overexpression lines despite GA accumulation. Together, findings suggest that *SlHsfC1* regulates tomato height via GA signaling.

### 4.2. SlHsfC1 Influences the Stature of Tomato Plants by Interacting with the Promoter Region of the Gibberellin SlGAI3 Signaling Gene

Because Hsfs can bind to HSE cis-elements, the DBD domain specifically recognizes the heat shock element (HSE, -GAAnnTTC-) and the stress response element (STRE, -AGGGG-) [30]. Given that *SlHsfC1*-overexpression lines exhibit a typical GA-independent phenotype [65], we focused on GA signal transduction genes that were upregulated in these lines and whose promoters contain HSE or STRE cis-elements. Collectively, results show SlHsfC1 binds the *SlGAI3* promoter and induces the expression of *SlGAI3*. Overexpression of *SlGAI3* leads to its binding to the GA signal pathway [21], thereby blocking GA signal transmission and adversely affecting the development and growth of tomato plants. A previous study found that suppression of *DELLA* genes induced dwarfism in tomato, whereas increased *DELLA* transcript levels did not visibly affect stem morphology [66]. DELLA proteins interact with various regulatory hubs to coordinate plant growth and stress adaptation [51]. In *Physcomitrium patens*, mutants lacking two *PpDELLA* genes did not show growth defects or altered responses to abiotic stress, possibly due to species-specific growth conditions. In contrast, *PpDELLA* positively regulates spore germination and sporophyte formation [63]. Transcriptomic findings have also been experimentally validated in *Marchantia polymorpha*. Analysis of the sole *DELLA*-encoding gene in this species indicates that *MpDELLA* accumulation counteracts oxidative stress, likely through increased production of flavonoids and other antioxidant compounds [67]. *MpDELLA* overaccumulation inhibits growth by limiting cell proliferation in the apical meristem [67].

*FaHsfC1b* overexpression increased survival, chlorophyll content, and photochemical efficiency while reducing electrolyte leakage and ROS under heat stress [31]. Additionally, HsfC1 can be induced by abiotic stresses such as low temperatures and drought, suggesting it plays a crucial role in stress resistance mechanisms [68,69,70]. A widely accepted explanation for the dwarfism observed in stress-tolerant plants is that stress-responsive genes may modulate *DELLA* activity. We propose that *SlHsfC1* regulates *SlGAI3* expression to influence plant height.

*SlHsfC1* overexpression unexpectedly caused severe dwarfism. Numerous previous studies have reported that abiotic stress-related genes can induce dwarfism [71,72]. The dwarf phenotype may also involve additional genes within hormone signaling pathways [73]. Exogenous IAA only partially restored growth in the overexpression lines (Figure S1). Based on GA content analysis (Figure 4c), we speculate that the GA signal transduction pathway is likely disrupted in these plants.

Unfortunately, we were unable to generate hybrid lines combining *SlGAI3* mutants and *SlHsfC1* overexpression lines. *SlHsfC1* also regulates gene expression of GA metabolism. *GA2ox1* could degrade active GA, the expression of *GA2ox1* in overexpressed plants increased, the expression level of knockout lines decreased, and the *GA2ox1* promoter −168 bp contained HSE elements, which may be the target gene of *SlHsfC1*, which needs to be verified by subsequent experiments. In production practice, elucidating the dwarfing mechanism of *SlHsfC1* enables tomato plants to grow normally under abiotic stress conditions without hindering their growth and development, thereby extending the tomato cultivation cycle. Consequently, the overexpression lines could not be functionally restored, limiting the ability to assess their stress tolerance phenotype. To address this issue, we constructed the pBinary *Hsp70pro*-*SlHsfC1* vector to produce heat-inducible transgenic plants. In these lines, *SlHsfC1* is not expressed under normal temperature conditions, enabling us to evaluate its functional role specifically in response to heat stress.

## 5. Conclusions

This study investigates HsfC originates from angiosperms, and there are double-ended repeats in the HsfC gene in the Solanaceae family. *SlHsfC1* localizes to the nucleus and responds to GA, SA, JA, and ethylene treatments. The phenotypic consequences of *SlHsfC1* overexpression include dwarfism and developmental retardation. A key finding was that active GA levels, particularly GA_1_ and GA_3_, showed elevated levels in *SlHsfC1* overexpression lines and did not allow rescue of dwarfism by GA3 application. Meanwhile, long-term GA_3_ treatment did not restore growth in overexpressing plants, and flowering and fruiting were adversely affected. Using RNA-Seq, RT–qPCR, EMSA, dual-luciferase assays, and yeast one-hybrid (Y1H) analysis, we identified *SlGAI3* as a downstream target of *SlHsfC1*. *SlHsfC1* overexpression upregulates *SlGAI3,* inducing GA insensitivity and dwarfism in tomato.

## Data Availability

The original contributions presented in this study are included in the article/Supplementary Material. Further inquiries can be directed to the corresponding author. The RNA-Seq results were submitted on the NCBI (BioProject: PRJNA1362956).

## Supplementary Materials

The following supporting information can be downloaded at https://www.mdpi.com/article/10.3390/plants14233617/s1, Figure S1. Phenotypic comparison of SlHsfC1-overexpressing lines and wild-type (AC) plants under IAA treatment (+IAA) or mock treatment (+mock). One-month-old tomato seedlings were sprayed with either 100 μM exogenous IAA or water one time at 3-day intervals. Figure S2. Long-term GA3 treatment of overexpressing lines resulted in abnormal plant architecture. Figure S3. Quantification of Gibberellin Levels in SlHsfC1 Crispr-cas9 Line. Values are mean ± standard deviation, Student’s *t*-test (n = 3). * *p* < 0.05. ** *p* < 0.01. *** *p* < 0.001. **** *p* < 0.0001; ns, no significant difference. Figure S4. RNA-Seq KEGG enrichment analysis. Figure S5. RNA-Seq GO enrichment analysis. Table S1. filter.annot.
