# Supplementary material for: The SlHsfC1–SlGAI3 Module Controls Tomato Growth and Development via the Gibberellin Signaling Pathway
Source: Plants (Basel). 2025 Nov 27;14(23):3617. doi: 10.3390/plants14233617 (PMC12693931; doi:10.3390/plants14233617)
Supplement: Supplementary file 1 [file plants-14-03617-s001.zip › Supplementary Figure.pdf]

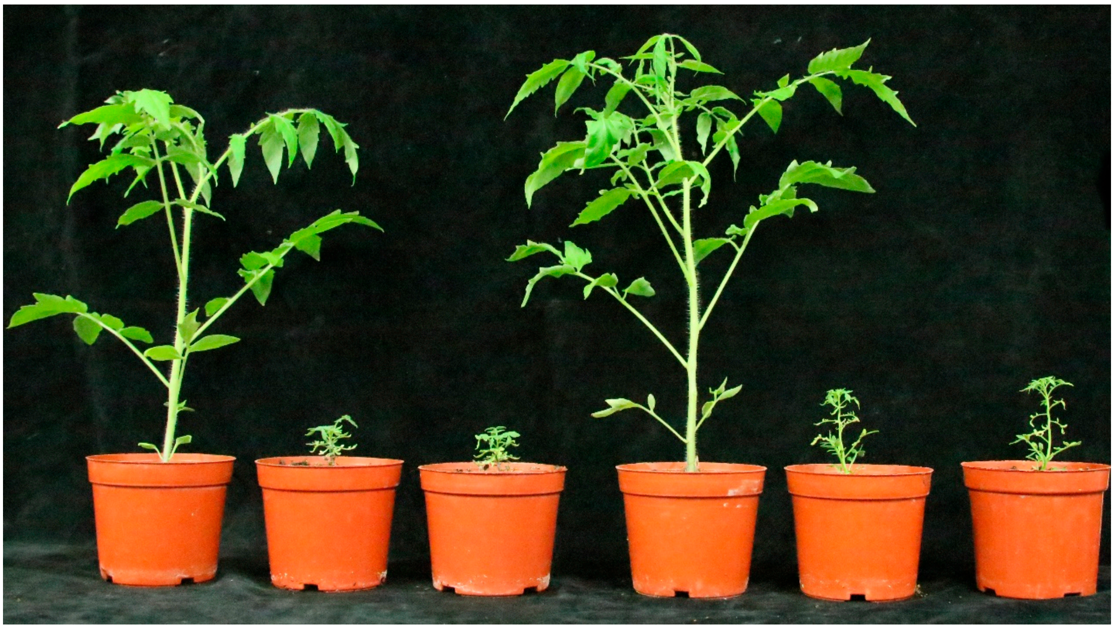

Figure S1. Phenotypic comparison of *SlHsfC1*-overexpressing lines and wild-type (AC) plants under IAA treatment (+IAA) or mock treatment (+mock). One-month-old tomato seedlings were sprayed with either 100 μM exogenous IAA or water one time at 3-day intervals.

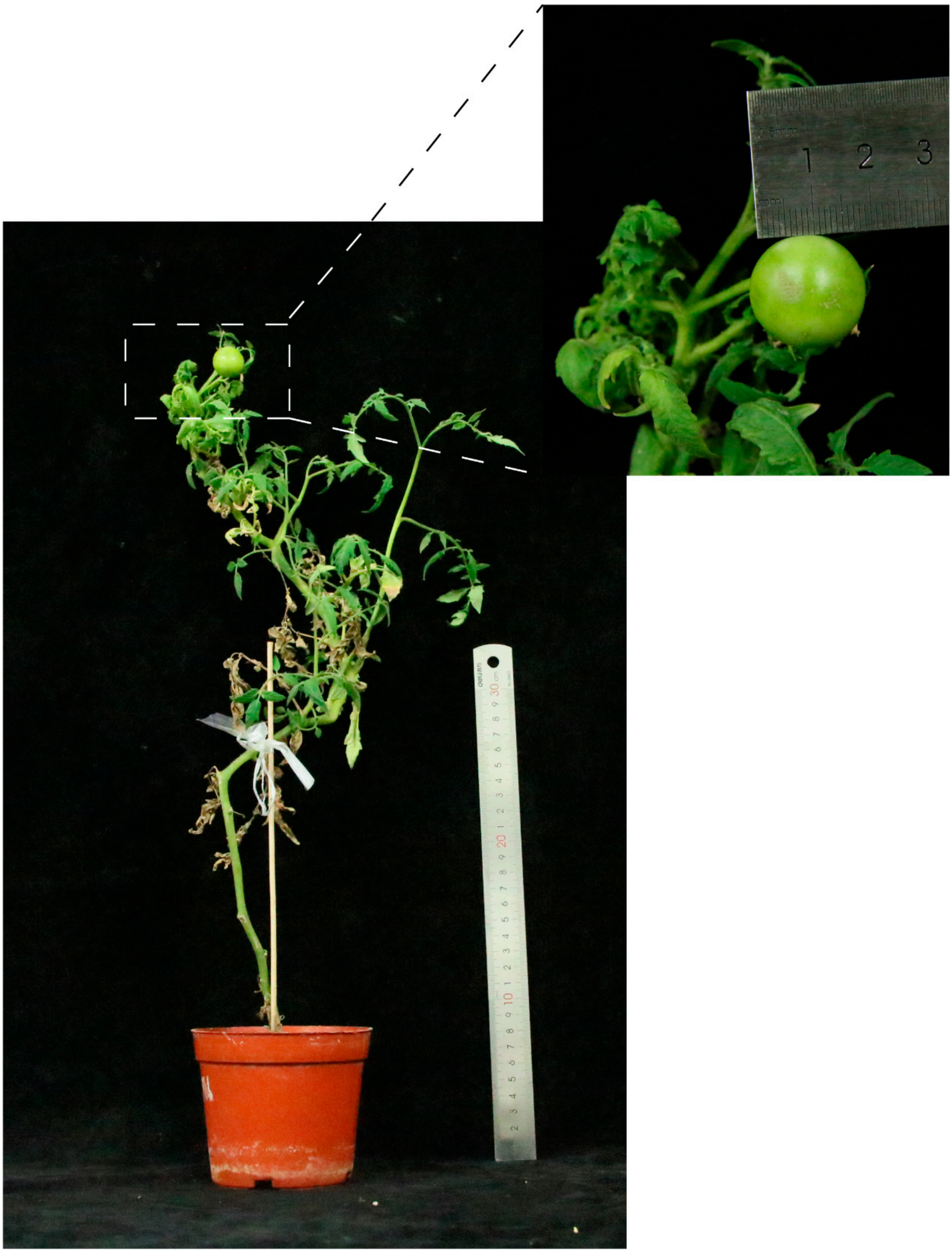

Figure S2. Long-term GA3 treatment of overexpressing lines resulted in abnormal plant architecture.

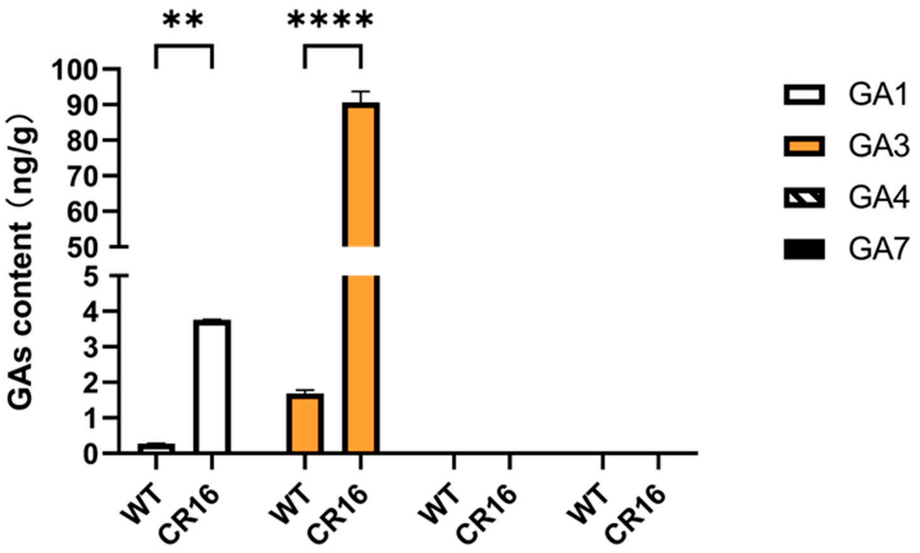

Figure S3. Quantification of Gibberellin Levels in *SlHsfC1* Crispr-cas9 Line. Values are mean ± standard deviation, Student's *t*-test ($n = 3$). $^{*}P < .05$. $^{**}P < .01$. $^{***}P < .001$. $^{****}P < .0001$; ns, no significant difference.

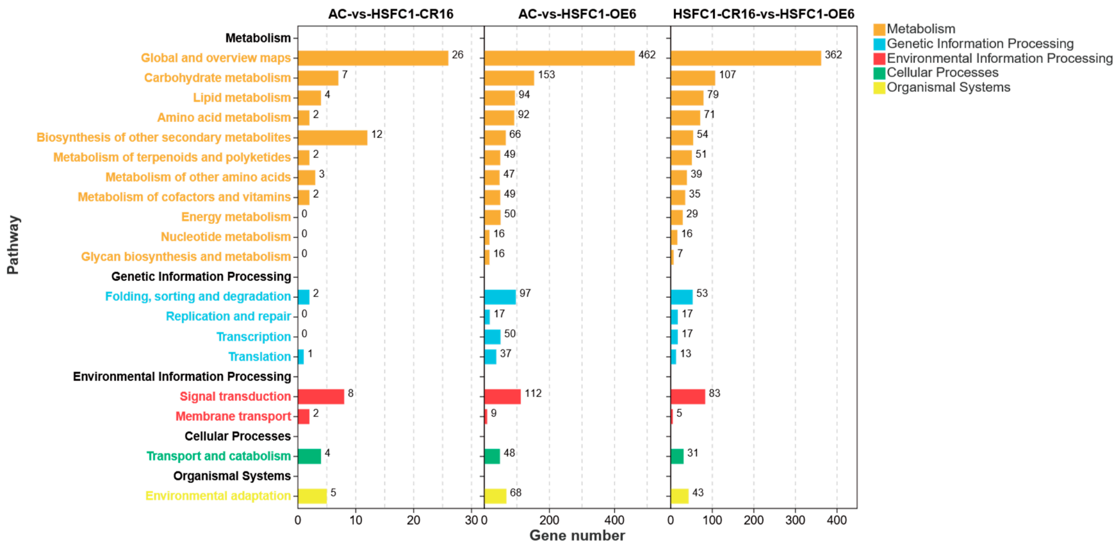

Figure S4. RNA-Seq KEGG enrichment analysis

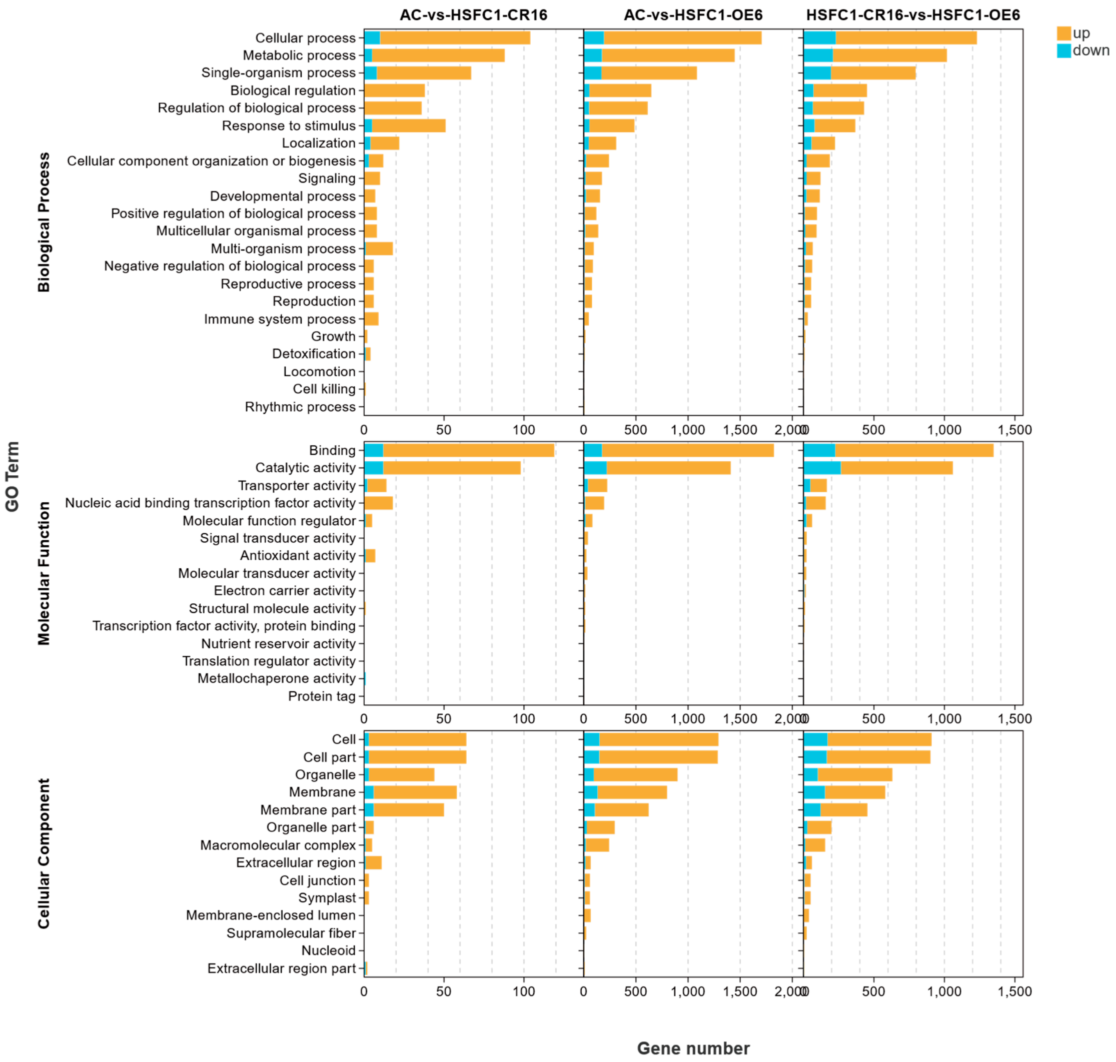

Figure S5. RNA-Seq GO enrichment analysis
